# Measuring accuracy of plethysmography based respiratory rate measurement using pulse oximeter at a tertiary hospital in India

**DOI:** 10.1186/s41479-020-00067-2

**Published:** 2020-06-05

**Authors:** Varun Alwadhi, Enisha Sarin, Praveen Kumar, Prasant Saboth, Ajay Khera, Sachin Gupta, Harish Kumar

**Affiliations:** 1 Kalawati Saran Children’s Hospital, C- 604, Connaught Circus, Bangla Sahib Rd, DIZ Area, Connaught Place, New Delhi, Delhi, 110001 India; 2 IPE Global Limited, B-84, Defence Colony, New Delhi, 110024 India; 3 Ministry of Health and Family Welfare, Nirman Bhavan, New Delhi, 110011 India; 4 USAID India Mission, American Embassy, Shantipath, Chanakyapuri, New Delhi, Delhi, 110021 India

**Keywords:** Under-five pneumonia, Respiratory rate, Pulse oximeter, Child health, India

## Abstract

**Background:**

Childhood pneumonia continues to be a major infectious killer in India. WHO recommended respiratory rate and oxygen saturation (SpO_2_) measurements are not well implemented in Indian public health outpatient facilities with the result that treatment decision-making rely on subjective assessments from variably trained and supervised healthcare providers. The introduction of a multi-modal pulse oximeter (POx) that gives reliable measurements would mitigate incorrect diagnosis. In light of future potential use of pulse oximeter in peripheral health centres, it becomes important to measure accuracy of respiratory rate and oxygen saturation of such an instrument. The current study measures accuracy of plethysmography based respiratory rate (RR) using a pulse oximeter (Masimo Rad-G) by comparing it with a gold standard (pediatrician) measurement.

**Study design:**

A cross sectional study was conducted in the OPD and emergency ward of Kalawati Saran Children’s Hospital over a 2 week period wherein a convenience sample of 97 children (2 to 59 months) were assessed by a pediatrician as part of routine assessment alongside independent measure by a consultant using pulse oximeter. The level of agreement between plethymography based RR and pediatrician measure was analyzed along with sensitivity and specificity of fast breathing of plethymography based RR measure.

**Results:**

Both methods of measurement show strong association (97%, *p* < 0.001) and observed values, falling on line of unity, obtained either from pulse oximeter or by pediatrician are very close to each other. Fast breathing measured by POx has a sensitivity of 95% and specificity of nearly 94%.

**Conclusion:**

The current study provides evidence of the accuracy of a plethysmography based RR using a pulse oximeter which can potentially be of use in planning of pneumonia management in public health facilities.

## Background

Globally, the number of episodes of clinical pneumonia in young children decreased by 22% from 178 million in 2000 to 138 million in 2015. Yet in the same year, India, Nigeria, Indonesia, Pakistan, and China contributed to more than 54% of all global pneumonia cases, with 32% of the global burden from India alone [1]. Childhood Pneumonia continues to be the topmost infectious killer among under-five children, contributing to 15% of under five deaths (Approximately 1.4 lakhs children) annually in India [2].

While over the past two decades the implementation of World Health Organization (WHO) Integrated Management for Childhood Illness (IMCI) guidelines during primary healthcare (PHC) in low-middle income countries (LMIC) has made substantial contributions to child mortality reductions, considerable residual mortality remains [2, 3]. WHO IMCI guidelines recommend counting respiratory rate for 1 min and checking SpO2 in sick children with cough or difficult breathing [4]. Recording respiratory rate and oxygen saturation (SpO2) are central to delivering high quality integrated care for sick infants and children, yet they are not well implemented in public health facilities in India, especially in peripheral health centres for a myriad of reasons including overburdened healthcare providers and non-availability of pulse oximeter.

Hypoxemia defined by WHO as a SpO2 < 90%, is associated with increased mortality in children [5]. As hypoxemia is cited as a key mortality risk factor among children with pneumonia, effectively identifying hypoxemic children is fundamental to reducing pediatric mortality globally, yet pulse oximetry (POx) is not available in primary health care (PHC) facilities. Modeling estimates of disease progression of pneumonia in the top 15 countries with the highest disease burden found that POx has the potential to avert 148, 000 deaths among children with pneumonia, and that, when combined with IMCI guidelines, the prognostic tool is highly cost-effective [6].

Many new ‘multi-modal devices’ that measure SpO2 as well as at least one other vital sign such as respiratory rate and/or temperature are in the developmental pipeline. Since few infants and children have these core vital signs collected, the end result of IMCI application at PHC is that referral and treatment decision-making rely on subjective assessments from variably trained and supervised healthcare providers. For example, in Malawi, it was found that of nearly 700 patient encounters with possible malaria or pneumonia only 16 and 24% had a respiratory rate (RR) or SpO2 measured. Most concerning was the observation that more than 40% of children eligible for hospitalization were not correctly referred [7]. A recent study in Malawi confirmed the potential utility and feasibility of POx at PHC. Malawian outpatient providers successfully measured SpO2 and found providers were two-fold more likely to correctly refer a child to the hospital when the SpO2 was low. Notably, > 60% of hypoxemic children would not have been referred to the hospital if providers had applied the 2014 WHO IMCI guidelines in the absence of a SpO2 measurement [8]. This premise can be extended to PHC where respiratory rate and SpO2 measurements are likely key barriers to optimal management of pneumonia. In fact, preliminary results of an assessment of pneumonia management practices among health workers conducted by the USAID supported Vriddhi project suggest a low awareness of the use of POx in the diagnosis of pneumonia except in the state of Haryana (internal report) [9]. In its absence, physical measurement of breathing rate and recognition of danger signs become essential in the diagnosis of ARI and/or pneumonia. Under the Ayushman Bharat scheme, sub centres and primary health centres (PHC) are being strengthened as Health and Wellness centers (HWC) to provide preventive and curative care for an expanded range of services including reproductive and child health services [10]. The services are to be provided by mid-level health care provider including Auxiliary Nurse Midwife (ANM), Ayush doctors, Community Health Officer (CHO) placed at a HWC and Medical Officer at PHC (Rural/Urban). However, we found that out of 45 providers in such centres, six reported not knowing how to measure RR and nine reported not knowing how to check labored breathing [9]. The introduction of POx would mitigate such issues, however, we would need a device which not only replaces clinical assessment but does so with a high level of precision.

It is within this overall context of improving the effectiveness of measuring the core vital signs of respiratory rate and SpO2 that we seek to evaluate a multi-modal device that records both. This is likely to have the added value of serving as a diagnostic for pneumonia and can help distinguish children with ARI into pneumonia who require referral or drugs or only home management with counselling. The reliability of pulse oximeter vis a vis other devices such as capnography has been studied, in which a high overall agreement was found with clinician reviewed capnography [11]. In addition, available evidence points to the accuracy of Masimo pulse oximeter in comparison with other pulse oximteres [12]. Pulse oximeters have been introduced in many newborn and pediatric care units in India, in tertiary health centres. With future plans to introduce it in the primary health care settings for diagnostic purpose in pneumonia, there is a need to investigate its reliability and add to the current knowledge. The current study compares clinician’s gold standard respiratory rate with device recorded respiratory rate based on plethysmography (Masimo Rad-G).

## Methods

This study compared respiratory rate measured through a pulse oxmeter (Masimo Rad-G) with the gold standard which we consider here as a pediatrician’s clinical measurement of respiratory rate. The study was conducted in the pediatric OPD and emergency unit of Kalawati Saran Hospital. The device used is the Masimo Rad-G which is a multimodal pulse oximter designed for use in pneumonia screening and spot checking of oxygen saturation in low resource settings. It uses measure-through Motion and Low Perfusion™ SET® pulse oximetry technology to measure SpO2, respiration rate from the Pleth (RRp™), pulse rate (PR), and perfusion index (Pi).

### Sample size

To determine the sample size, n, we used the relative error r and the difference between the overall agreement probability Pa and the chance-agreement probability Pe as follows

$$ \mathrm{n}=\left(\mathrm{n}\ast \right)/\left(1+\mathrm{n}\ast /\mathrm{N}\ \right)\kern2.5em \mathrm{n}\ast =1/\left(\mathrm{r}2\ \left(\mathrm{pa}-\mathrm{pe}\right)2\right) $$

The pulse oximeter, overall, provides an accurate measurement of respiratory rate and oxygen saturation compared with other devices. Therefore, we consider a low relative error between measurements (0.20), and an assumed level of agreement: 0.50 (as this is an unknown value). With the total population of children admitted to the pediatric ward at any given time being an average of 50 (N), we get a sample size of 50. We oversampled to include a total of 97 children as we aimed toward precision of the measures.

### Data collection

A convenience sample of 97 children between 2 months to 59 months were assessed over a period of 2 weeks by the pediatrician. Measurements were done at the first hospital contact when children were admitted to the OPD or emergency. Every day, 6–10 children were administered the POx – they were selected as and when they came into the OPD or the emergency ward. Children with shock, toe deformities or inconsolability were excluded. For every selected child, the pediatrician measured the respiratory rate and wrote it in a column of the data collection sheet. A study consultant (junior resident) then used the multimodal device on the child, took an independent measurement and wrote it against the previous column.

### Ethical clearance

The study did not require any ethical review as use of POx is a common practice in the children’s inpatient wards and is a standard practice management according to WHO IMCI guideline. The Masimo POx was used as it is a globally approved and standardized equipment.

### Statistical analysis

We used three different types of analysis to measure linear association between the POx derived RR and physician derived RR: 1) Pearson Correlation Coefficient, 2) Bland Altman method, and 3) Sensitivity and Specificity analysis.

Advanced new equipments and instruments used in medical diagnosis need a few essential assessment of measurement’s acceptability theory. We used two broad types of measurement errors i.e. systematic errors (Bias) and random errors (Standard Deviation). Bias is mean difference between measured (pulse oxymeter) and true values (pediatrician measurement reference as accuracy) while random error is expressed as the standard deviation of measured values. We also stated root mean square deviation (RMSD) that combines both mean bias and random error to give a total error measure, calculated as follows:

$$ \mathrm{RMSD}=\surd \left(\left[\sum \left\{\mathrm{RRPulse}\ \mathrm{oxymeter}-\mathrm{RRpediatrician}\right\}2\right]/\mathrm{n}\right), $$

**Fig. 1:**
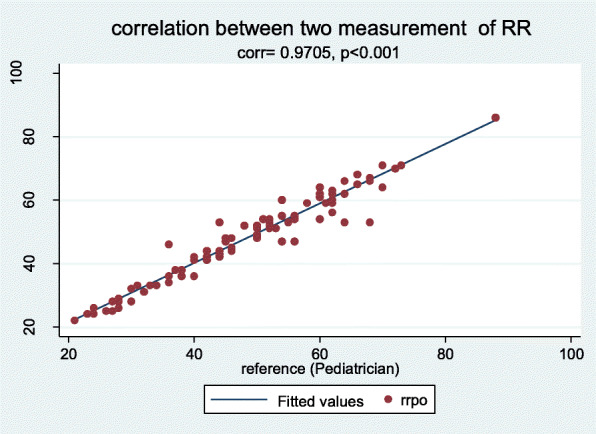
Correlation between RR values obtained from both the measurements

Further, Pearson correlation coefficient is represented as an indication of linear association between the measured and reference at *p* < 0.001. A linear fit and line of unity fit was tested to observe the data appropriateness for linearity (Fig. 1) Another test of association Lin’s concordance correlation coefficient was obtained as a single-magnitude summary of agreement. We assumed a normal approximation interval (α = .05) to compute the 95% confidence interval (95% CI) for these coefficients.

Additionally, analysis by components of variance technique was followed, as described by Bland and Altman. This method compensates for multiple measurements being taken from the same subject when the true reference value is changing. A modified Bland–Altman diagram was used to graphically represent the data, displaying mean bias and limits of agreement. Here, the mean of the 2 independent observations was replaced by one of the reference measurements. That is, the reference is assumed to have negligible error and so forms the x-axis of the plot. We assumed a normal approximation of limit of agreement of measuring device (α = 0.05) to compute the 95% confidence interval (95% CI) for these coefficients. Further, sensitivity and specificity of pulse oximeter RR over reference was statistically analyzed. Clinically, fast breathing is recorded as more than or equal to 50 for 2 to 12 months old infants whereas for children above 12 months, it is more than or equal to 40. RR diagnosis result of children obtained from pediatrician was taken as reference hence RR measured by advance equipment Pulse Oxymeter was compared with this existing method (Table 2).

Analyses were performed using STATA 12.0 version.

## Results

Total 97 subjects were obtained under study and all the data were analyzed. Selected demographic characteristics and respiratory diagnosis results using two measurements are presented in Table 1. About 75% children were male and average age of children was 15 months. Out of total 97 children, the majority (72%) were diagnosed to be suffering from pneumonia, most with some associated complications including TB and SAM while the rest were admitted for some form of sepsis, abscess, cellulitis and anemia. Average RR observed using POx was a bit lower (48.1) than reference measure (48.8). Average systematic error of measurement was observed by Pulse Oximeter less than 0 (0.40, SD = 3.31) i.e. on average test measurement showed lower values than reference results. Overall divergence from the reference measurement was found to be about 3 (RMSD) (Table 1).

**Table 1:** Characteristics of sampled children under study (*N* = 97)

Variables	Mean	SD	Min	Max
**Demographic**				
Age (months)	15.31	12.3	2.5	54
Gender (M/F)	74/23			
**Respiratory Diagnosis**				
SPO2	94.92	4.5	80	100
RR oximeter	48.41	13.3	22	86
RR reference	48.81	13.7	21	88
Bias (Pulse Oximeter –True value@Reference)	−0.40	3.31	−15	10
Random Error (SD)	13.3			
RMSD (root mean square deviation)	3.31			
**Test of agreement**	**Correlation value**		**UCL**	**LCL**
Pearson Correlation	0.970**	0.001		
Concordance correlation coefficient	0.976**	0.006	0.958	0.981
95% limits of agreement (for difference)	−0.40(mean)	3.309	6.084	−6.888

*UCL* upper confidence limit, *LCL* Lower confidence limit

**Fig. 2:**
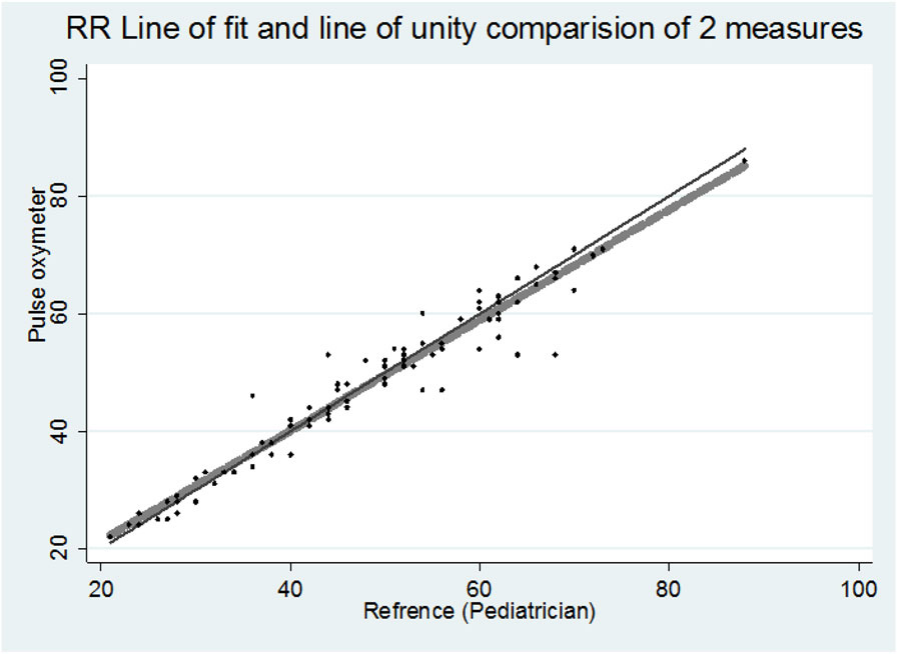
Line of fit of RR values obtained from both the measurements

**Fig. 3:**
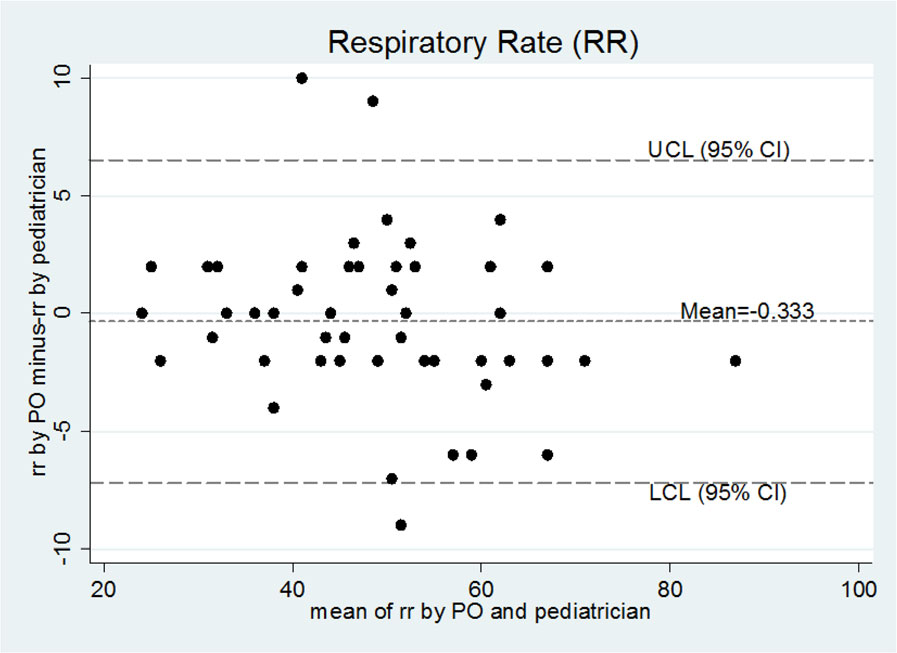
Bland and Altman Plot for difference in two measurements vs mean

Both methods of measurement on RR shows significant strong association (97%) over the RR values (Fig. 2) at 0.1% level of significance (*p* < 0.001) and again line of Linear fit on observed values lies on line of unity, hence, values obtained either from Pulse Oximeter or By Pediatrician (Gold Standard) are very close to each other (Fig. 3). While, the Lin’s concordance correlation coefficient indicates an overall agreement of 0.967 (0.958, 0.981) (Table 1).

From the sampled data, 95% limits of agreement (mean bias ±1.96 × SD) were 6.08 and − 6.89 breaths per minute. Hence the results measured by method POx are 7 units below or 6 units above method of pediatrician (Table1, Fig. 3).

High sensitivity (95%) and specificity (94%) of fast breathing as detected by POx with 95% of accuracy was obtained. There is 95% probability of a diagnostic test using POx that a child is correctly identified as having pneumonia. Similarly, higher specificity represents the 94% probability of a test diagnosis of a child without fast breathing who will be identified as negative in this study. Additionally, a considerably high level of agreement (Kappa = 0.85) was found between the POx and reference values of fast breathing and the difference in values is by chance alone (Table 2). The Masimo POx, thus, allows for significantly integrated result of RR and SPO2 with high sensitivity and accuracy in health care settings, as well as the possibility of rapid detection.

**Table 2:** Sensitivity and Specificity of RR of Pulse Oximeter

Statistic	Value (%)	95% CI
Sensitivity	95.38	87.10 to 99.04
Specificity	93.75	79.19 to 99.23
Accuracy	94.85	88.38 to 98.31
Kappa	0.847	0.81 to 0.88

## Discussion

The results show a high level of agreement between pleth based RR using a POx and physician measured RR, indicating a high accuracy of RR reading of the POx. Accurate measurement of respiratory rate is an essential step toward correct diagnosis of pneumonia [13]. RR is recognized as an important risk marker in community acquired pneumonia [14]. In the absence of doctors, front line health workers screen and diagnose diseases. In a study in Zambia, despite moderately high level of agreement between CHW classification of children with fast and normal breathing with that of experts, the limits of agreement were wide enough to lead the authors to conclude that further use of diagnostic tools such as POx would greatly help in assessing pneumonia in remote settings in LMIC [15].

On the other hand, there have been studies where inter observer agreement was found to be poor in RR measurements [16, 17]. Variability was found even when measurements were performed using WHO recommended methods leading the authors to suggest development of objective technological solutions [16]. Evidence shows POx to give the most accurate and objective measurement to identify hypoxemia in children and WHO recommends it as a standard diagnostic tool [13].

In our study, pleth based RR using a POx had a high level of agreement with that of the gold standard, indicating a high level of accuracy. The sensitivity analysis, in addition, points to the reliability of the device in correctly identifying fast breathing, a major symptom of the disease, in 95% of the cases. Using it in primary health care centers by staff who are not clinically qualified, like ANM and AYUSH doctors would strengthen the pneumonia management program by facilitating correct diagnosis of cases of pneumonia. Currently, health care workers are trained on IMCI guidlelines, the effect of which has been mixed- while a low evidence was found on fewer deaths among children from birth to 5 years in a Cochrane review, there was insubstantial evidence on the performance of health care workers in treating common illnesses [18]. Another review found that in 13 of 21 studies, health workers prescribed incorrect medicines, thus highlighting the need for correct diagnosis and prescription [19]. In our own project assessment, only 24% of ANM, CHO, and AYUSH doctors in health and wellness centers had complete knowledge of how to measure respiratory rate and recognizing all the danger signs, while only 11% had complete knowledge of the IMCI based signs of labored breathing [9]. The introduction of a reliable and accurate instrument in the diagnosis of pneumonia would not only ease the diagnosis process, but is expected to lead to simplified IMCI guidelines in pneumonia management. In the absence of clinicians, health workers diagnose pneumonia based on chest indrawing and fast breathing which is difficult to measure. The use of a multi-modal device could potentially simplify algorithms for pneumonia case management. Our study has limitations as the two measures were administered simultaneously- one after another, and the data collectors were not blinded, which may potentially carry recording bias. Another limitation is that we conducted the study in a tertiary hospital but we wish to carry the implication of the findings on to periphery facilities which has different levels and quality of infrastructure. Nevertheless, we believe that the evidence generated from a real life clinical situation has potential value in the application of pulse oximeter with pleth based RR measurement in periphery centres for pneumonia screening and management.

## Conclusion

There is a high degree of agreement between pleth based RR using a POx and physician measured RR, indicating that the former provides reliable and accurate measurement. Current diagnosis and management of pneumonia in primary health care is based on variably trained health providers despite IMCI guidelines. The use of pulse oximeter, also recommended by WHO, which can provide reliable measurement would streamline pneumonia case management in these settings. The current study provides evidence of the reliability of a pulse oximeter.

## Data Availability

The raw data will be made available upon request.

## Abbreviations

POx: Pulse Oximeter
RR: Respiratory Rate
PHC: Primary health care
IMCI: Integrated Management for Childhood Illness
WHO: World Health Organization
LMIC: Low and Middle Income Countries
Spo2: peripheral capillary oxygen saturation
TB: Tuberculosis
SAM: Severe Acute malnutrition
USAID: United States Agency for International Development
ANM: Auxilliary Nurse Midwife
AYUSH: Ayurvedic, Yoga and Naturopathy, Unani, Siddha and Homeopathy
CHO: Community Health Officer
OPD: Outpatient Department
